# Hamilton Early Warning Score: predict, prevent and protect

**DOI:** 10.1186/cc14581

**Published:** 2015-03-16

**Authors:** B Tam, M Xu, AE Fox-Robichaud

**Affiliations:** 1 McMaster University, Hamilton, ON, Canada

## Introduction

This study determined the pattern of decline prior to an inpatient arrest. We implemented the Hamilton Early Warning Score (HEWS) within our electronic vital signs documentation to track and trigger care for deteriorating patients. Other EWS have been described in the literature with varying success [1]. In contrast to previous observational studies, we chose to implement a score modified from published EWS using the consensus opinion of a steering committee and evaluate the score in real time.

## Methods

We conducted a prospectively identified, retrospectively gathered cohort study at two hospitals of consecutively admitted medical and surgical patients over a 6-month period. One hospital had a rapid response team (RRT) and used HEWS with a trigger of 5 while the other was undergoing implementation of the HEWS without a RRT. HEWS was calculated for each patient on the first day of admission and for the 3 days prior to inpatient arrest or death. A study investigator reviewed all events for accuracy. Our outcome of interest was a composite of inpatient cardiac arrest and hospital mortality.

## Results

**Figure 1:**
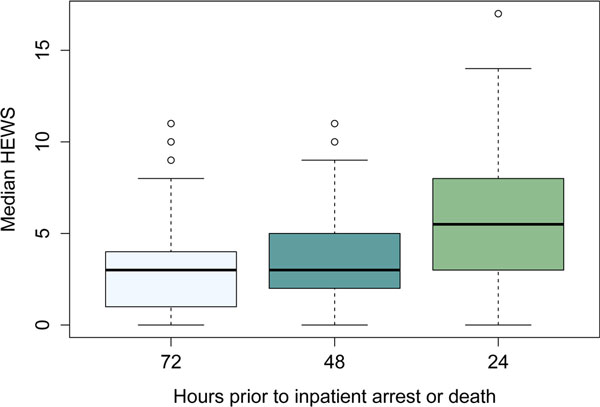
**HEWS prior to critical event**.

There were 7,138 patients admitted over 6 months. We found 0.5% of patients suffered an inpatient arrest and 3.6% of patients died. Moreover, 66% of patients who died or arrested were admitted to the hospital without a RRT. Patients who arrested or died had more comorbidities defined by the Charlson Comorbidity Index (CCI) of 6.0 and 8.2 respectively compared with the general population, which had a CCI of 5.2. The median and mean HEWS at time of admission was 1 and 1.7 for the general population, 2 and 2.4 for patients who suffered an inpatient arrest and finally 3 and 3.8 for those who died. There was a rise in median HEWS from 2 to 5 in the 24 hours prior to in patient arrest or death. See Figure 1.

## Conclusion

We found that a 2.5-fold increase in HEWS occurred 24 hours prior to critical events. Similar to previous studies, a RRT in conjunction with HEWS is the best system to reduce unanticipated adverse events. An absolute HEWS of 5 and/or a rapidly increasing HEWS should trigger rapid assessment and treatment to reduce preventable inpatient deaths and arrests.
